# Determinants of thrombocytopenia in critically ill patients: a systematic review and meta-analysis

**DOI:** 10.3389/fmed.2026.1833527

**Published:** 2026-07-09

**Authors:** Xiaoming Su, Xuefang Ben, Yanjie Tian

**Affiliations:** 1 Department of Cardiac Surgery, Affiliated Hospital of Hebei University, Baoding, China; 2 Outpatient Department, Beidaihe Rest and Recuperation Center, Qinhuangdao, China; 3 The 82nd Group Army Hospital of the People's Liberation Army, Baoding, China

**Keywords:** critically ill patients, intensive care unit, meta-analysis, risk factors, thrombocytopenia

## Abstract

**Background:**

Thrombocytopenia frequently occurs in critically ill patients and is associated with adverse clinical outcomes in the intensive care unit (ICU). Although numerous studies have explored potential contributors, the factors associated with the development of thrombocytopenia in critically ill populations have not been consistently summarized. This study aimed to synthesize available evidence and identify determinants associated with thrombocytopenia in critically ill patients.

**Methods:**

A systematic search of PubMed, Embase, Web of Science, the Cochrane Library, CNKI, Wang fang and VIP were performed from database inception to 20 January 2026. Observational studies reporting factors associated with thrombocytopenia in critically ill patients were considered eligible. Two reviewers independently screened studies and extracted relevant data. The methodological quality of the included studies was evaluated using the Newcastle–Ottawa Scale (NOS). Meta-analysis was conducted using Stata15 software, and pooled estimates were calculated using a random-effects model.

**Results:**

A total of 13 cohort studies involving 27,147 critically ill patients were included, among whom 4,820 developed thrombocytopenia. The pooled analysis indicated that bleeding was associated with a higher occurrence of thrombocytopenia (OR = 3.49, 95% CI: 1.70–7.19). A similar association was observed for sepsis (OR = 2.32, 95% CI: 1.74–3.09). Impaired liver function was also linked to an increased likelihood of thrombocytopenia (OR = 1.55, 95% CI: 1.14–2.11). In addition, a Simplified Acute Physiology Score (SAPS) < 20 showed a modest association with thrombocytopenia (OR = 1.09, 95% CI: 1.05–1.14).

**Conclusion:**

Bleeding, sepsis, impaired liver function, and lower SAPS scores were associated with a higher likelihood of thrombocytopenia among critically ill patients. These findings highlight several clinical conditions that frequently accompany platelet decline in ICU settings and may assist clinicians in identifying patients who require closer monitoring. Additional prospective research may help clarify the underlying mechanisms and further define the clinical implications of these associations.

## Background

Thrombocytopenia is a common hematological abnormality in critically ill patients and occurs in a substantial proportion of individuals admitted to the intensive care unit (ICU) (1). Previous studies have shown that platelet decline during critical illness is associated with greater disease severity and worse clinical outcomes, including prolonged ICU stay, longer duration of mechanical ventilation, and increased mortality (2–4). Because platelet counts are routinely monitored in ICU practice, thrombocytopenia may provide clinically useful information regarding the progression of systemic illness and the emergence of complications.

The mechanisms underlying thrombocytopenia in critically ill patients are complex and often multifactorial. Platelet decline may occur in the setting of infection, bleeding, coagulopathy, organ dysfunction, and treatment-related exposures, among other conditions (5, 6). However, the determinants reported across individual studies have varied considerably, partly because of differences in patient populations (7), ICU settings, thrombocytopenia definitions, and statistical adjustment strategies. As a result, the available evidence remains fragmented, and the relative strength and consistency of reported associations are still not well defined (8, 9).

Although several observational studies have explored potential predictors of thrombocytopenia in ICU populations, most were conducted in single centers or specific subgroups of critically ill patients, and their findings have not been systematically integrated in a quantitative manner. Therefore, the present systematic review and meta-analysis was conducted to synthesize available observational evidence on determinants associated with thrombocytopenia in critically ill patients. By quantitatively pooling data across studies, this review aimed to identify the clinical factors most consistently associated with thrombocytopenia, provide a clearer framework for risk assessment in ICU practice, and highlight methodological issues that should be addressed in future prospective research.

## Methods

### Study design and reporting guidelines

This systematic review and meta-analysis were conducted in accordance with the Preferred Reporting Items for Systematic Reviews and Meta-Analyses (PRISMA) guidelines (10). The protocol was registered in the International Prospective Register of Systematic Reviews (PROSPERO; CRD420261343235). The study aimed to synthesize available evidence regarding factors associated with thrombocytopenia in critically ill patients admitted to intensive care units.

### Literature search strategy

A comprehensive literature search was performed in PubMed, Embase, Web of Science, the Cochrane Library, CNKI, Wang fang and VIP from database inception to 20 January 2026. The search strategy combined controlled vocabulary terms and free-text keywords related to thrombocytopenia and critically ill patients. The main search terms included “thrombocytopenia,” “critically ill,” “intensive care unit,” “ICU,” “risk factors,” and “predictors.” Boolean operators (AND/OR) were used to combine search terms. In addition, the reference lists of relevant studies and reviews were manually screened to identify additional eligible articles. The detailed search strategy is provided in Supplementary Table S1.

### Eligibility criteria

Studies were considered eligible if they met the following criteria:

- Observational studies (cohort studies, case–control studies, or cross-sectional studies).
- Conducted in critically ill patients admitted to the ICU.
- Reported potential determinants or risk factors associated with thrombocytopenia.
- Provided sufficient data to calculate effect estimates, such as odds ratios (ORs) with corresponding confidence intervals (CIs). This study has no language restrictions.
- Studies were excluded if they:
- Were case reports, reviews, conference abstracts, or editorials.
- Did not report relevant outcomes related to thrombocytopenia.
- Included overlapping or duplicated patient populations.

### Study selection and data extraction

All retrieved records were first screened by two reviewers independently based on titles and abstracts. Studies that appeared potentially relevant were subsequently assessed through full-text review according to the predefined inclusion and exclusion criteria. Any disagreements between reviewers were resolved through discussion, and if necessary, consultation with a third reviewer. Data extraction was conducted independently by two investigators using a standardized form. The following information was collected from each eligible study: first author, year of publication, country, study design, sample size, number of thrombocytopenia, gender, mean age, definition of thrombocytopenia, regression model.

### Quality assessment

The methodological quality of the included observational studies was evaluated using the Newcastle–Ottawa Scale (NOS) (11). This tool assesses study quality based on three domains: selection of study groups, comparability of groups, and ascertainment of exposure or outcomes. The total score ranges from 0 to 9 points, with higher scores indicating better methodological quality. Quality assessment was performed independently by two reviewers, and discrepancies were resolved through consensus.

### Statistical analysis

All statistical analyses were performed using Stata15 software. Effect sizes were expressed as ORs with corresponding 95%CIs. Considering the potential clinical and methodological differences among the included studies, a random-effects model was applied in all analyses. Statistical heterogeneity was assessed using the I^2^ statistic and the Cochran *Q* test. Sensitivity analyses were conducted by sequentially removing individual studies to evaluate the stability of the pooled estimates. Potential publication bias was examined using funnel plots and Egger’s regression test, and when publication bias was suggested, the trim-and-fill method was used to explore its possible impact on the pooled results.

## Results

**Figure 1:**
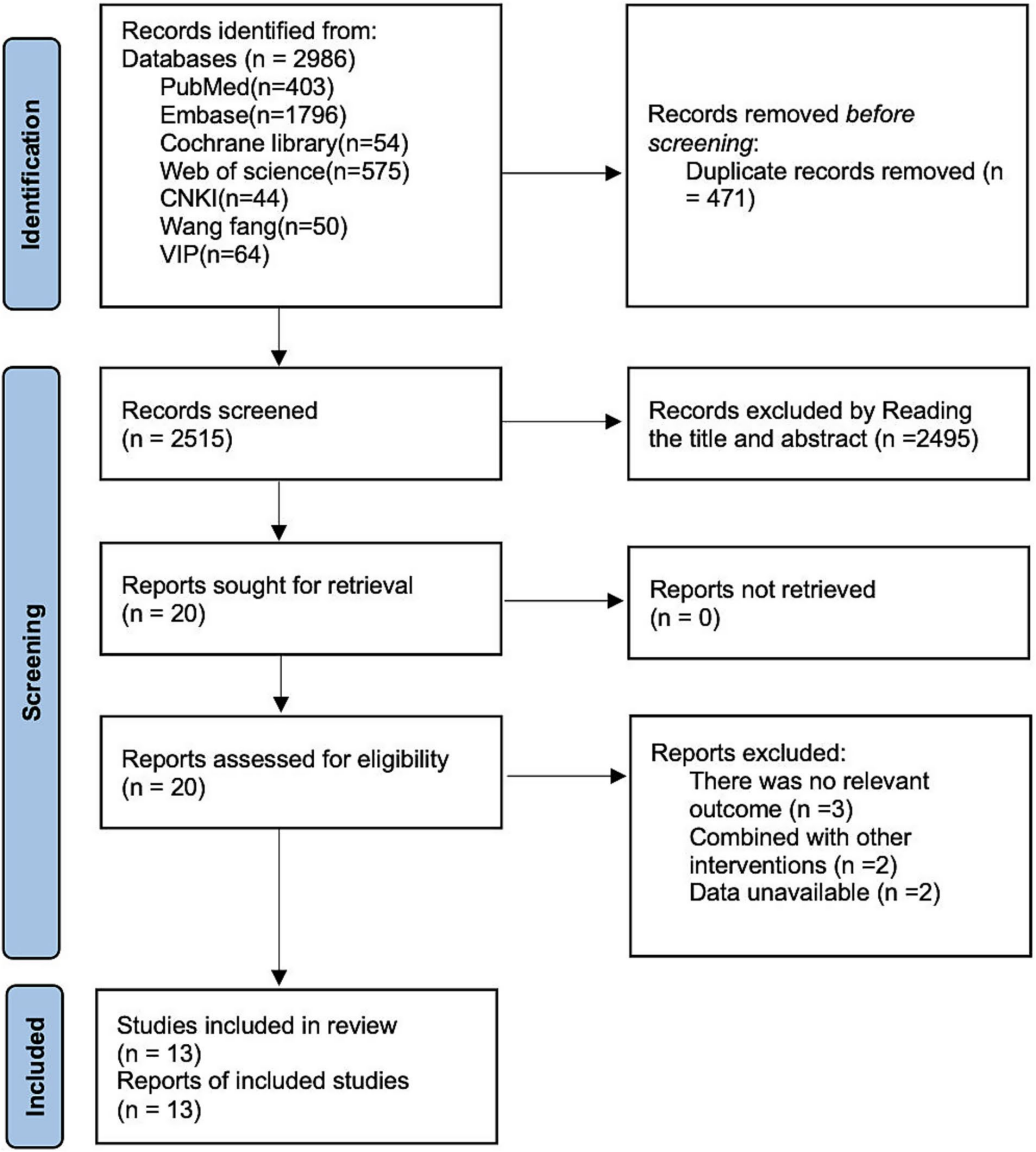
Flow diagram of the study selection process.

A total of 2,986 records were retrieved through searches of seven electronic databases, including PubMed (*n* = 403), Embase (*n* = 1,796), the Cochrane Library (*n* = 54), Web of Science (*n* = 575), CNKI (*n* = 44), Wan fang (*n* = 50), and VIP (*n* = 64). After the removal of 471 duplicate entries, 2,495 articles were excluded because they were clearly unrelated to the study objective. 7 studies were excluded by reading the full article. Finally, 13 studies (12–24) satisfied the predefined inclusion criteria and were included in the meta-analysis. The overall study selection procedure is illustrated in Figure 1.

### Characteristics of the included studies

The characteristics of the included studies are presented in Table 1. A total of 13 cohort studies published between 2005 and 2026 were included, involving 27,147 critically ill patients, of whom 4,820 developed thrombocytopenia. The studies were conducted in several countries, including China, Canada, Spain, Greece, Morocco, Saudi Arabia, and Egypt. Sample sizes varied considerably across studies, ranging from 95 to 20,696 participants. The mean age of participants ranged from 50 to 84 years, indicating that most populations consisted of older critically ill patients. Male patients accounted for a slightly higher proportion than females in most studies. Thrombocytopenia was primarily defined as a platelet count <150 × 10^9^/L, although several studies used a threshold of <100 × 10^9^/L. All included studies employed multivariate logistic regression models to identify factors associated with thrombocytopenia.

**Table 1:** Characteristics of the studies included in the meta-analysis.

Study	Year	Study design	Country	Sample size	Number of thrombocytopenia	Gender (M/F)	Mean age (years)	Diagnostic criteria for thrombocytopenia	Regression model
Aissaoui	2007	Cohort study	Morocco	112	41	67/45	50	Platelet count < 150 × 10^9^/L	Multivariate logistic regression
Almardod	2026	Cohort study	Saudi Arabia	276	112	167/109	63	Platelet count < 150 × 10^9^/L	Multivariate logistic regression
Cheng	2025	Cohort study	China	3,147	1,009	1,779/1,368	74	Platelet count < 100 × 10^9^/L	Multivariate logistic regression
Crowther	2005	Cohort study	Canada	261	121	156/105	66.9	Platelet count < 150 × 10^9^/L	Multivariate logistic regression
Ebid	2025	Cohort study	Egypt	422	168	181/241	63	Platelet count < 150 × 10^9^/L	Multivariate logistic regression
Marco	2012	Cohort study	Spain	587	37	379/208	69	Platelet count < 150 × 10^9^/L	Multivariate logistic regression
Papakitsou	2024	Cohort study	Greece	249	102	131/118	82.3	Platelet count < 150 × 10^9^/L	Multivariate logistic regression
Shalansky	2022	Cohort study	Canada	362	68	229/133	63.2	Platelet count < 150 × 10^9^/L	Multivariate logistic regression
Williamson	2013	Cohort study	Canada	20,696	2,755	10,666/10,030	64.2	Platelet count < 150 × 10^9^/L	Multivariate logistic regression
XL Xu	2021	Cohort study	China	468	123	309/177	68.3	Platelet count < 100 × 10^9^/L	Multivariate logistic regression
T NI	2019	Cohort study	China	188	128	113/75	64.3	Platelet count < 100 × 10^9^/L	Multivariate logistic regression
J Sun	2014	Cohort study	China	95	45	59/36	65	Platelet count < 100 × 10^9^/L	Multivariate logistic regression
QX Liao	2020	Cohort study	China	324	111	206/118	84	Platelet count < 150 × 10^9^/L	Multivariate logistic regression

### Quality assessment

The detailed scoring results are shown in Table 2. Overall, the quality of the included studies was considered acceptable, with total scores ranging from 6 to 9 points. Five studies achieved the highest score of 9, indicating relatively strong methodological rigor, while four studies obtained 7 points and the remaining four studies scored 6 points.

**Table 2:** Quality assessment of the included studies using the Newcastle–Ottawa Scale (NOS).

Cohort study									
Study	Representativeness of the exposed group	Selection of non-exposed groups	Determination of exposure factors	Identification of outcome indicators not yet to be observed at study entry	Comparability of exposed and unexposed groups considered in design and statistical analysis	Design and statistical analysis	Adequacy of the study’s evaluation of the outcome	Adequacy of follow-up in exposed and unexposed groups	Total scores
Aissaoui 2007 (12)	*	*	*	*	**	*	*	*	9
Almardod 2026 (13)	*	*	*	/	**	*	*	*	8
Cheng 2025 (14)	*	*	*	/	**	*	*	*	8
Crowther 2005 (15)	*	*	*	*	**	*	*	*	9
Ebid 2025 (16)	*	*	*	*	**	*	*	*	9
Marco 2012 (17)	*	*	*	/	*	*	*	*	7
Papakitsou 2024 (18)	*	*	*	/	/	*	*	*	6
Shalansky 2022 (19)	*	*	*	/	*	*	*	*	7
Williamson 2013 (20)	*	*	*	/	/	*	*	*	6
Xu 2021 (21)	*	*	*	/	/	*	*	*	6
Ni 2019 (22)	*	*	*	/	*	*	*	*	7
Sun 2014 (23)	*	*	*	/	/	*	*	*	6
Liao 2020 (24)	*	*	*	/	*	*	*	*	7

*means one scores; **means two scores.

Most studies performed well in the selection domain, particularly with respect to the representativeness of the exposed cohort and the assessment of exposure. Outcome evaluation and follow-up were also generally adequate across the included studies. However, several studies received lower scores in the comparability domain, mainly because adjustment for potential confounding variables was limited in the study design or statistical analysis. Despite these limitations, the overall methodological quality of the included studies was considered sufficient for inclusion in the quantitative synthesis.

### Meta-analysis results

#### Bleeding

**Figure 2:**
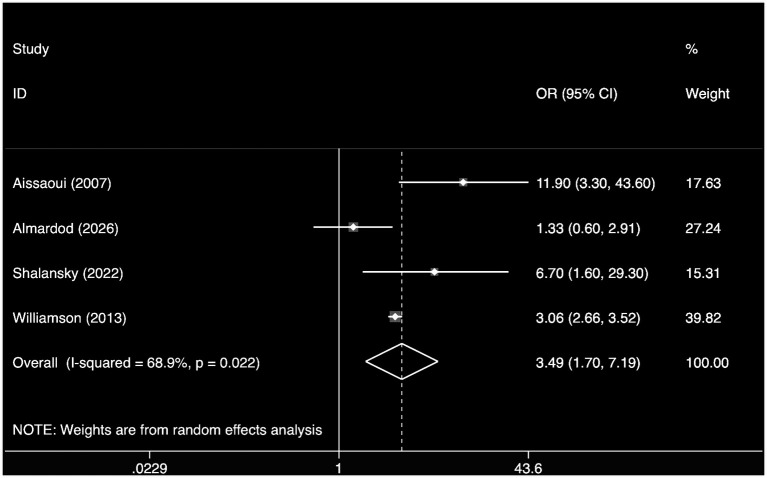
Association between bleeding and thrombocytopenia in critically ill patients.

The association between bleeding and thrombocytopenia was examined in four studies. Considerable between-study variability was observed (*I*^2^ = 68.9%, *p* = 0.022). Despite this heterogeneity, the pooled estimate derived from the random-effects model suggested that bleeding was associated with a higher likelihood of thrombocytopenia (OR = 3.49, 95% CI: 1.70–7.19) (Figure 2). Sequential omission of individual studies (Supplementary Figure S1) did not substantially affect the pooled estimate, suggesting stable results.

#### Sepsis

**Figure 3:**
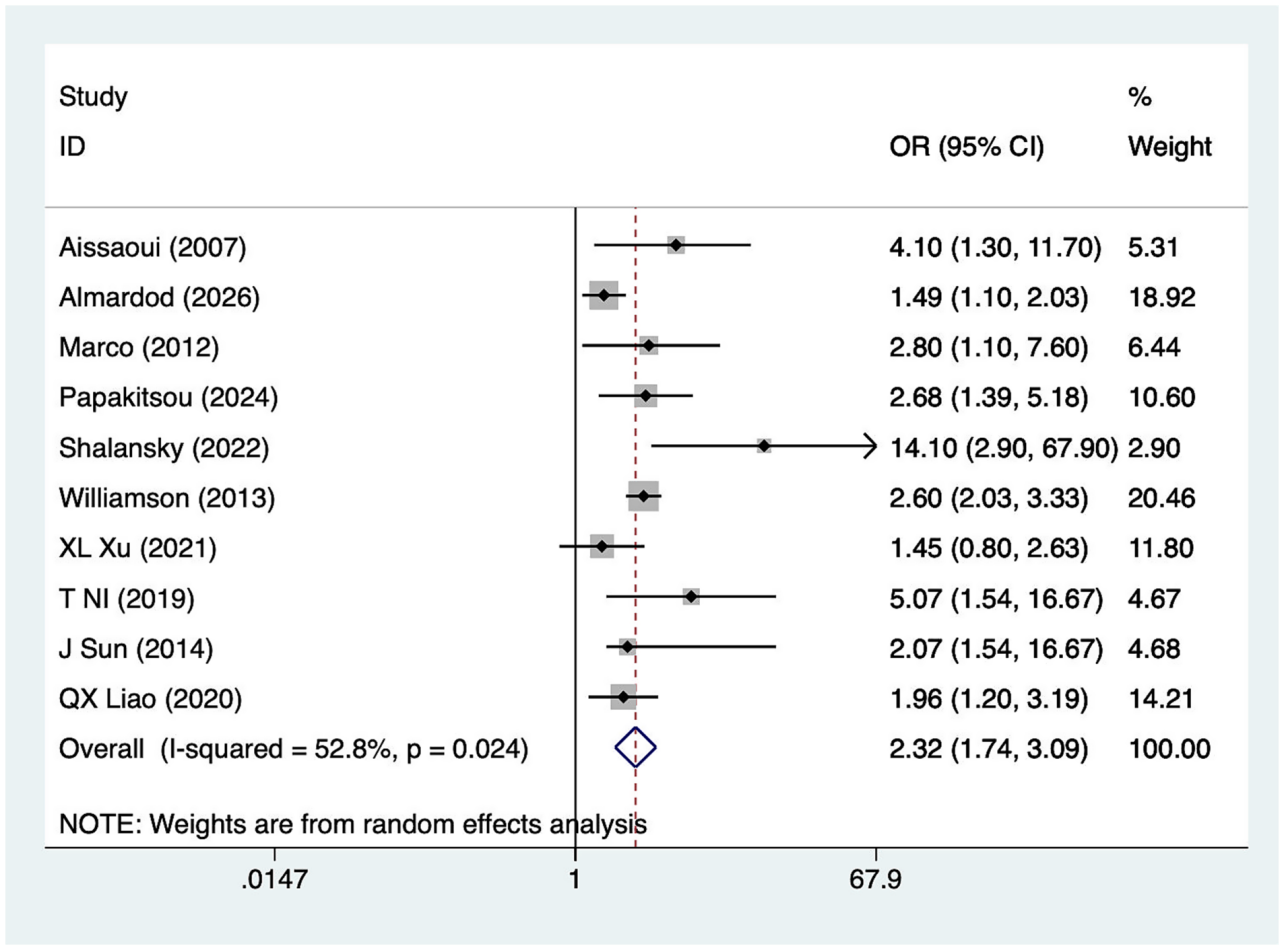
Association between sepsis and thrombocytopenia in critically ill patients.

The association between sepsis and thrombocytopenia was examined in 10 studies. Considerable between-study variability was observed (*I*^2^ = 52.8%, *p* = 0.024). Despite this heterogeneity, the pooled estimate derived from the random-effects model suggested that sepsis was associated with a higher likelihood of thrombocytopenia (OR = 2.32, 95% CI: 1.74–3.09) (Figure 3). Sequential omission of individual studies (Supplementary Figure S2) did not substantially affect the pooled estimate, suggesting stable results.

#### Simplified Acute Physiology Score (SAPS) < 20

**Figure 4:**
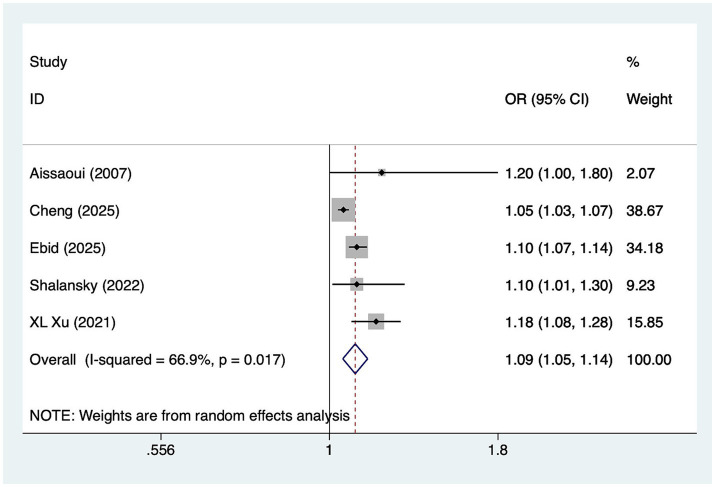
Association between Simplified Acute Physiology Score (SAPS) < 20 and thrombocytopenia.

Five studies explored the relationship between a SAPS < 20 and thrombocytopenia in critically ill patients. Considerable variability among studies was observed (*I*^2^ = 66.9%, *p* = 0.017). The pooled analysis based on the random-effects model suggested that a SAPS score below 20 was associated with an increased likelihood of thrombocytopenia (OR = 1.09, 95% CI: 1.05–1.14) (Figure 4). Sensitivity analysis (Supplementary Figure S3) showed that removing individual studies sequentially did not materially alter the pooled estimate, indicating stable results.

#### Impaired liver function

**Figure 5:**
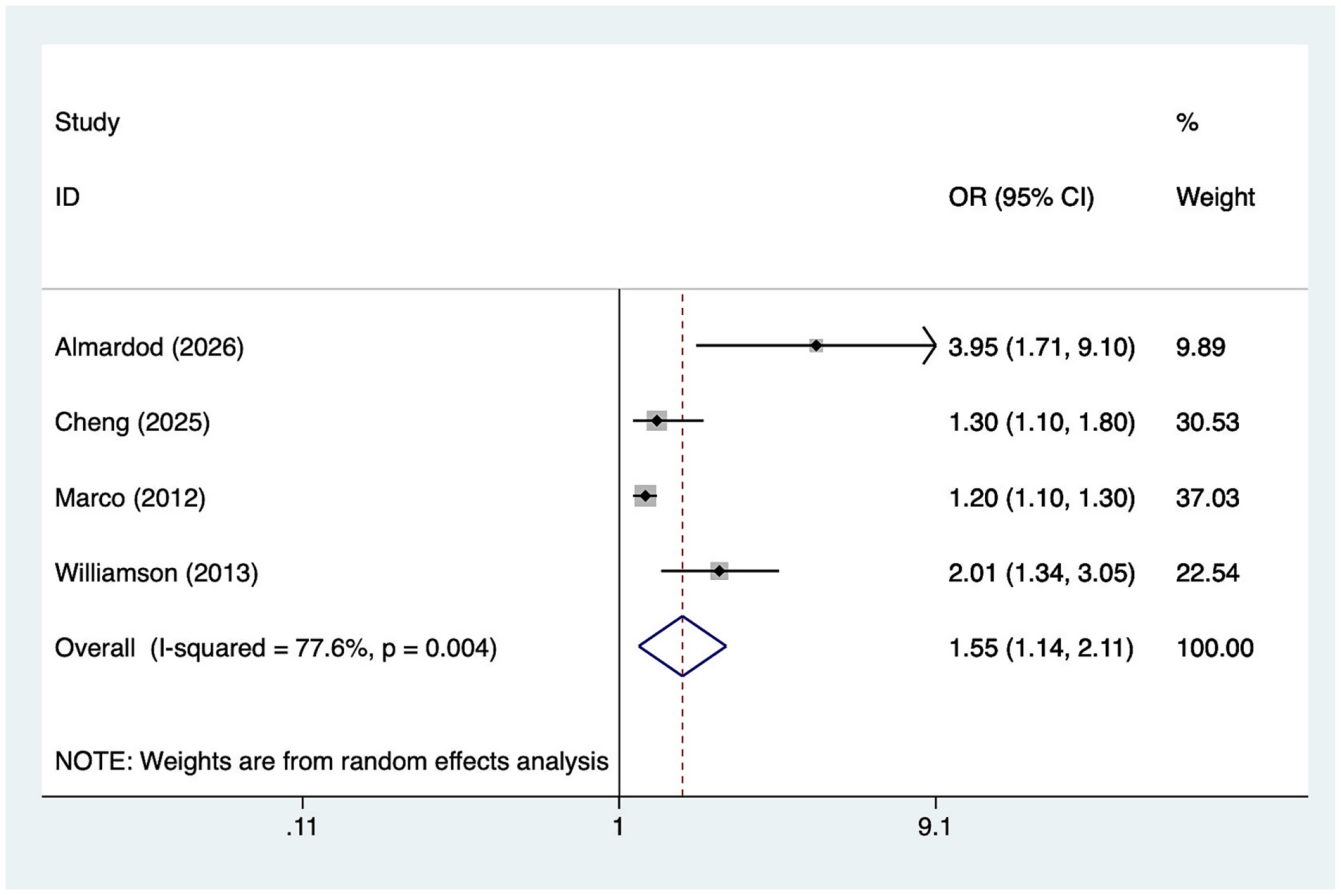
Association between impaired liver function and thrombocytopenia.

Four studies provided data on the relationship between impaired liver function and thrombocytopenia among critically ill patients. Variation between studies was evident (*I*^2^ = 77.6%, *p* = 0.004). After pooling the available estimates using a random-effects approach, impaired liver function was associated with a greater occurrence of thrombocytopenia (OR = 1.55, 95% CI: 1.14–2.11) (Figure 5). Sensitivity analysis (Supplementary Figure S4) yielded comparable estimates after the exclusion of individual studies, indicating that the overall finding was not dependent on a single dataset.

#### Subgroup analysis

**Figure 6:**
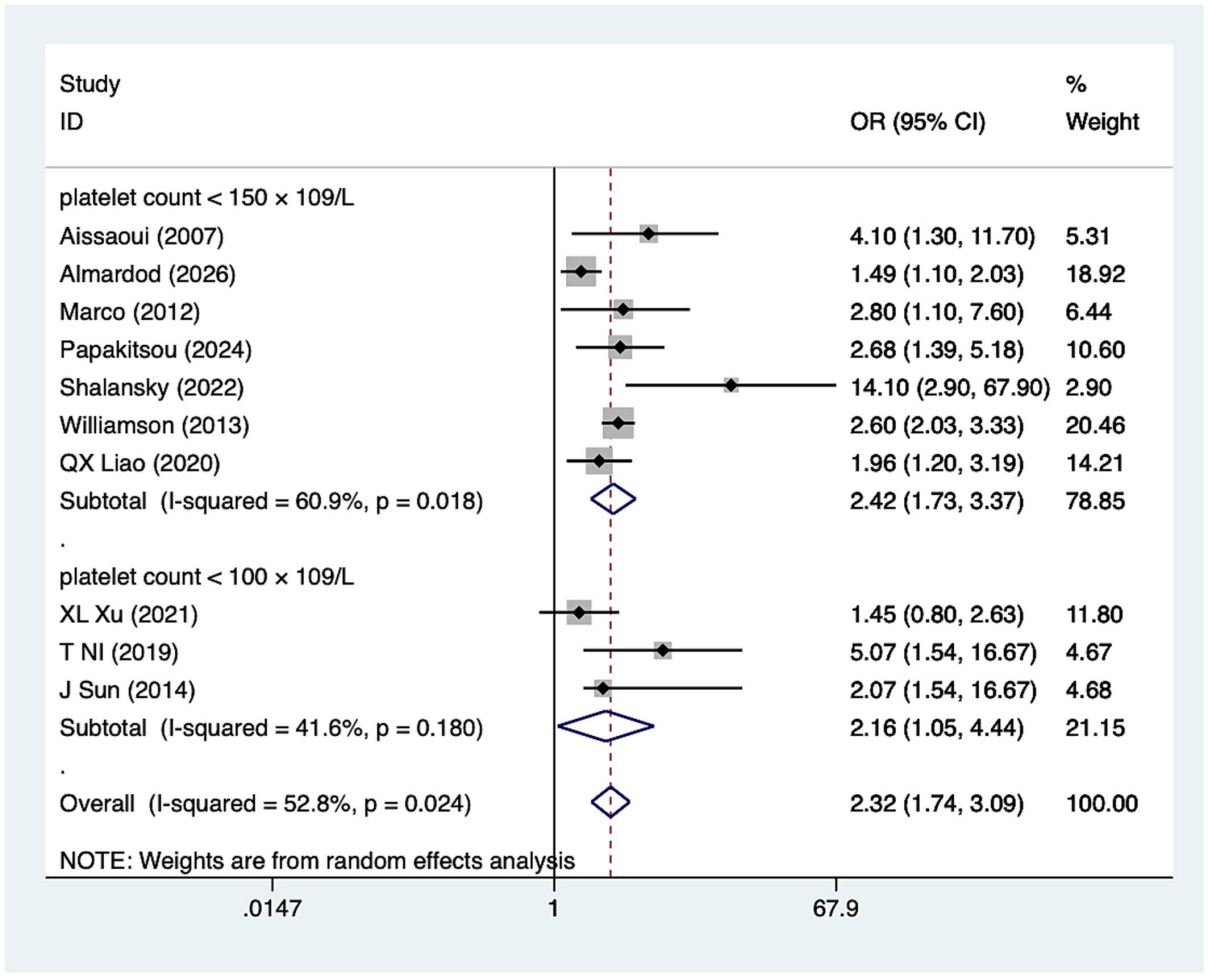
Subgroup analysis of sepsis and thrombocytopenia by platelet cut-off definition.

A subgroup analysis based on the thrombocytopenia threshold showed that sepsis was significantly associated with thrombocytopenia in both subgroups (Figure 6). In studies defining thrombocytopenia as platelet count <150 × 10^9^/L, the pooled OR was 2.42 (95% CI: 1.73–3.37; *I*^2^ = 60.9%). In studies using a stricter definition of platelet count <100 × 10^9^/L, the pooled OR was 2.16 (95% CI: 1.05–4.44; *I*^2^ = 41.6%). The similar direction and magnitude of the pooled estimates indicate that the observed association between sepsis and thrombocytopenia was not materially altered by differences in diagnostic thresholds.

#### Publication bias

Potential publication bias was examined using funnel plots together with Egger’s regression test. As shown in the Supplementary Figures S5–S8, the funnel plots for bleeding (Egger’s *p* = 0.795), sepsis (Egger’s *p* = 0.214), and SAPS < 20 (Egger’s *p* = 0.180) appeared largely symmetrical, suggesting no evident publication bias for these analyses. In contrast, the distribution of studies assessing impaired liver function showed noticeable asymmetry (Egger’s *p* = 0.03), raising the possibility of publication bias. To explore its potential influence, the trim-and-fill procedure was applied. After adjustment (Supplementary Figure S9), the pooled estimate remained comparable to the original result, indicating that the overall conclusion was unlikely to be substantially affected by potential publication bias.

## Discussion

In this systematic review and meta-analysis of 13 cohort studies involving 27,147 critically ill patients, bleeding, sepsis, impaired liver function, and lower SAPS were all associated with a higher likelihood of thrombocytopenia. These findings should be interpreted as associations rather than causal effects, but they help identify the clinical contexts in which platelet decline is more likely to occur during critical illness. Overall, the results support the view that thrombocytopenia in ICU populations is not an isolated hematologic abnormality, but rather a marker that frequently accompanies severe systemic disturbances.

Among the examined determinants, sepsis showed a consistent association with thrombocytopenia. This finding is biologically plausible and aligns with current understanding of the interaction between inflammation, coagulation, and platelet turnover during severe infection (25, 26). In septic states, systemic inflammatory activation may promote platelet consumption, endothelial injury, and microvascular thrombosis, while at the same time impairing platelet production through bone marrow suppression or dysregulated hematopoiesis. In ICU practice, thrombocytopenia is therefore often interpreted within the broader context of sepsis-related organ dysfunction rather than as an isolated laboratory abnormality (27).

Bleeding was also associated with a markedly greater likelihood of thrombocytopenia. This relationship is likely bidirectional and should be interpreted with caution. On the one hand, active bleeding may contribute to platelet consumption and dilutional effects, particularly in patients receiving fluid resuscitation or transfusion support (28, 29). On the other hand, reduced platelet counts may themselves increase susceptibility to hemorrhagic complications, especially in the presence of coagulopathy or severe systemic illness. For this reason, the observed association should not be understood as evidence of a simple one-way mechanism, but rather as reflecting the close clinical interplay between thrombocytopenia, hemostatic imbalance, and disease severity in critically ill patients (30).

Impaired liver function was another factor associated with thrombocytopenia in the pooled analysis. This association is also clinically plausible, as the liver plays a central role in thrombopoietin production and in the regulation of coagulation pathways (31, 32). Hepatic dysfunction may reduce thrombopoietin synthesis, contribute to platelet sequestration, and coexist with coagulopathy or systemic inflammation, all of which may favor platelet decline. In critically ill patients, liver dysfunction is often not an isolated process but part of broader multiorgan impairment, which may further amplify its relationship with thrombocytopenia (33).

The association between lower SAPS and thrombocytopenia was statistically significant but relatively modest in magnitude, and its interpretation deserves caution. Severity scores such as the SAPS are composite indices designed to summarize the overall physiological condition of critically ill patients rather than represent a single biological pathway (34, 35). Accordingly, the association observed here may indicate that thrombocytopenia tends to occur in patients with greater overall physiological derangement, rather than suggesting a direct mechanistic link between the score itself and platelet decline. This finding should therefore be viewed mainly as supportive evidence that thrombocytopenia often emerges in the setting of broader clinical deterioration (36).

An important contribution of the present review is that it moves beyond isolated single-center observations and provides a quantitative synthesis of multiple clinically relevant determinants across ICU populations. Rather than focusing on a single exposure or a specific subgroup, this meta-analysis integrates the available observational evidence into a broader framework that identifies which clinical conditions show the most consistent associations with thrombocytopenia. In this respect, the value of the review lies less in proposing a novel biomarker and more in consolidating fragmented evidence into a more clinically interpretable summary that may inform monitoring strategies and future study design.

Several limitations should be considered. First, all included studies were observational, and residual confounding cannot be excluded despite multivariable adjustment in many studies. Second, heterogeneity was substantial in several pooled analyses, which may reflect differences in patient characteristics, ICU case mix, study design, covariate adjustment, and clinical practice patterns across centers. Third, the definition of thrombocytopenia was not fully consistent across studies, with some using a platelet threshold of <150 × 10^9^/L and others using <100 × 10^9^/L. This variation may have influenced comparability and may partly explain the observed between-study heterogeneity. Fourth, some pooled analyses were based on a relatively small number of studies, limiting the precision and robustness of factor-specific estimates. Finally, potential publication bias could not be excluded for the impaired liver function analysis, although the trim-and-fill analysis did not materially change the pooled result.

From a clinical perspective, these findings suggest that thrombocytopenia should be interpreted as a warning sign that may accompany severe infection, bleeding, organ dysfunction, and overall physiological instability. Because platelet counts are routinely available in ICU settings, awareness of the clinical conditions most associated with platelet decline may help clinicians identify patients who warrant closer monitoring and more careful diagnostic evaluation. However, the current evidence does not support causal inferences, nor does it establish that these associated factors can be used in isolation for prediction or treatment decisions.

Future prospective studies with standardized diagnostic thresholds for thrombocytopenia, more consistent adjustment for confounders, and clearer reporting of ICU-specific clinical variables are needed to strengthen the evidence base. Large multicenter cohorts may also help clarify how infection, bleeding, organ dysfunction, and severity of illness interact over time to influence platelet dynamics. Such work would improve comparability across studies and may support more refined approaches to monitoring thrombocytopenia in critically ill patients.

## Conclusion

This meta-analysis suggests that bleeding, sepsis, impaired liver function, and lower SAPS were all associated with a greater occurrence of reduced platelet counts in ICU populations. These patterns suggest that thrombocytopenia in critically ill patients often arises within a broader context of systemic physiological disturbances, particularly those related to infection, organ dysfunction, and hemostatic imbalance.

Recognition of these associated factors may help clinicians better interpret platelet declines and identify patients who require closer monitoring during intensive care management. Nevertheless, the evidence summarized in this analysis is derived from observational studies, and residual confounding cannot be fully excluded. Further well-designed prospective studies with standardized diagnostic criteria may help clarify the mechanisms underlying thrombocytopenia and strengthen the clinical implications of these associations.

## Data Availability

The original contributions presented in the study are included in the article/Supplementary material, further inquiries can be directed to the corresponding author.
