# Supplementary material for: Determinants of thrombocytopenia in critically ill patients: a systematic review and meta-analysis
Source: Front Med (Lausanne). 2026 Jul 9;13:1833527. doi: 10.3389/fmed.2026.1833527 (PMC13431168; doi:10.3389/fmed.2026.1833527)
Supplement: Supplementary file 1 [file Data_Sheet_1.docx]

Table S1 detailed search strategy

((("Intensive Care Units"[Mesh]) OR ((((Intensive Care Units[Title/Abstract]) OR (Intensive Care Unit[Title/Abstract])) OR (Unit, Intensive Care[Title/Abstract])) OR (ICU Intensive Care Units[Title/Abstract]))) AND (("Thrombocytopenia"[Mesh]) OR ((((Thrombocytopenia[Title/Abstract]) OR (Thrombocytopenias[Title/Abstract])) OR (Thrombopenia[Title/Abstract])) OR (Thrombopenias[Title/Abstract])))) AND (("Risk Factors"[Mesh]) OR (((((((((((((((((((Risk Factors[Title/Abstract]) OR (Factor, Risk[Title/Abstract])) OR (Risk Factor[Title/Abstract])) OR (Population at Risk[Title/Abstract])) OR (Populations at Risk[Title/Abstract])) OR (Risk Scores[Title/Abstract])) OR (Risk Score[Title/Abstract])) OR (Score, Risk[Title/Abstract])) OR (Risk Factor Scores[Title/Abstract])) OR (Risk Factor Score[Title/Abstract])) OR (Score, Risk Factor[Title/Abstract])) OR (Health Correlates[Title/Abstract])) OR (Correlates, Health[Title/Abstract])) OR (Social Risk Factors[Title/Abstract])) OR (Factor, Social Risk[Title/Abstract])) OR (Factors, Social Risk[Title/Abstract])) OR (Risk Factor, Social[Title/Abstract])) OR (Risk Factors, Social[Title/Abstract])) OR (Social Risk Factor[Title/Abstract])))


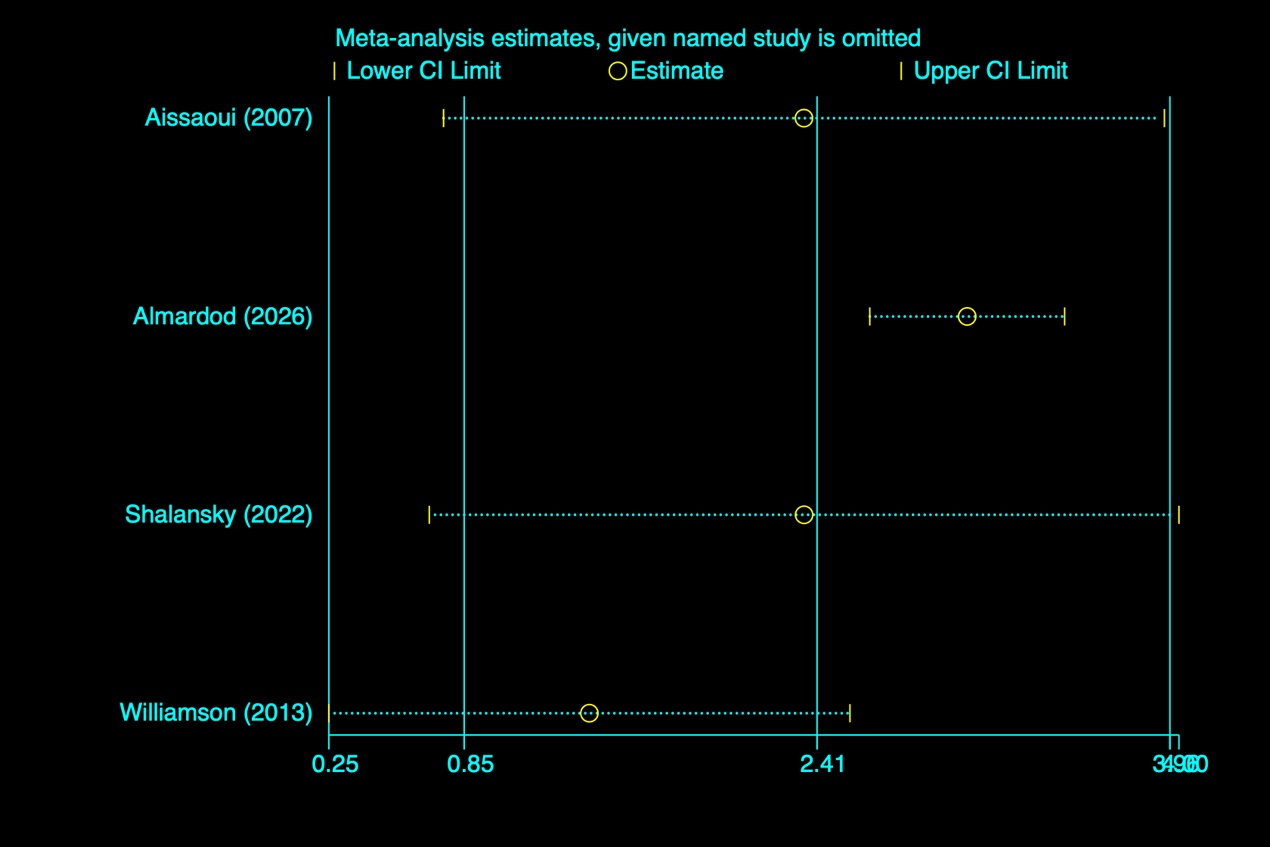


Figure S1 sensitivity analysis results of bleeding


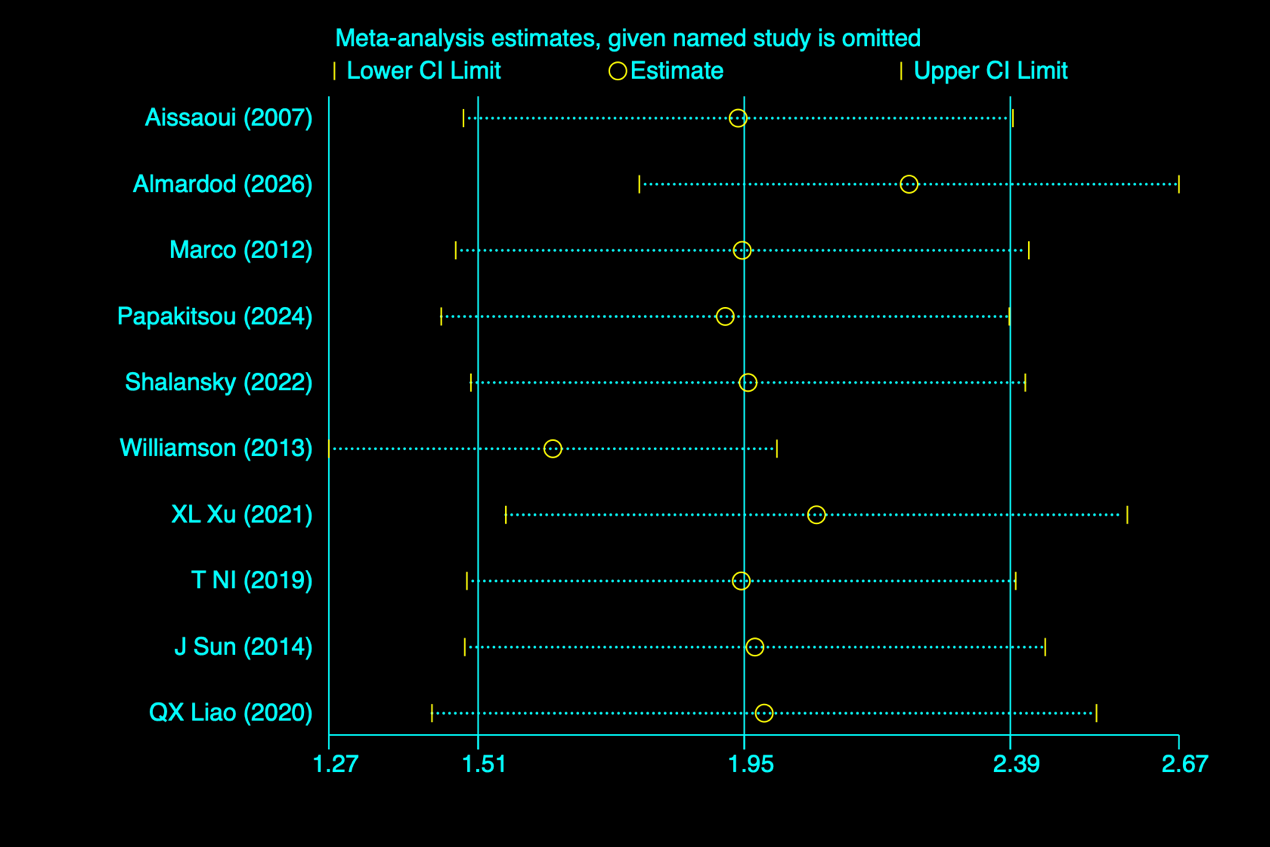


Figure S2 sensitivity analysis results of sepsis


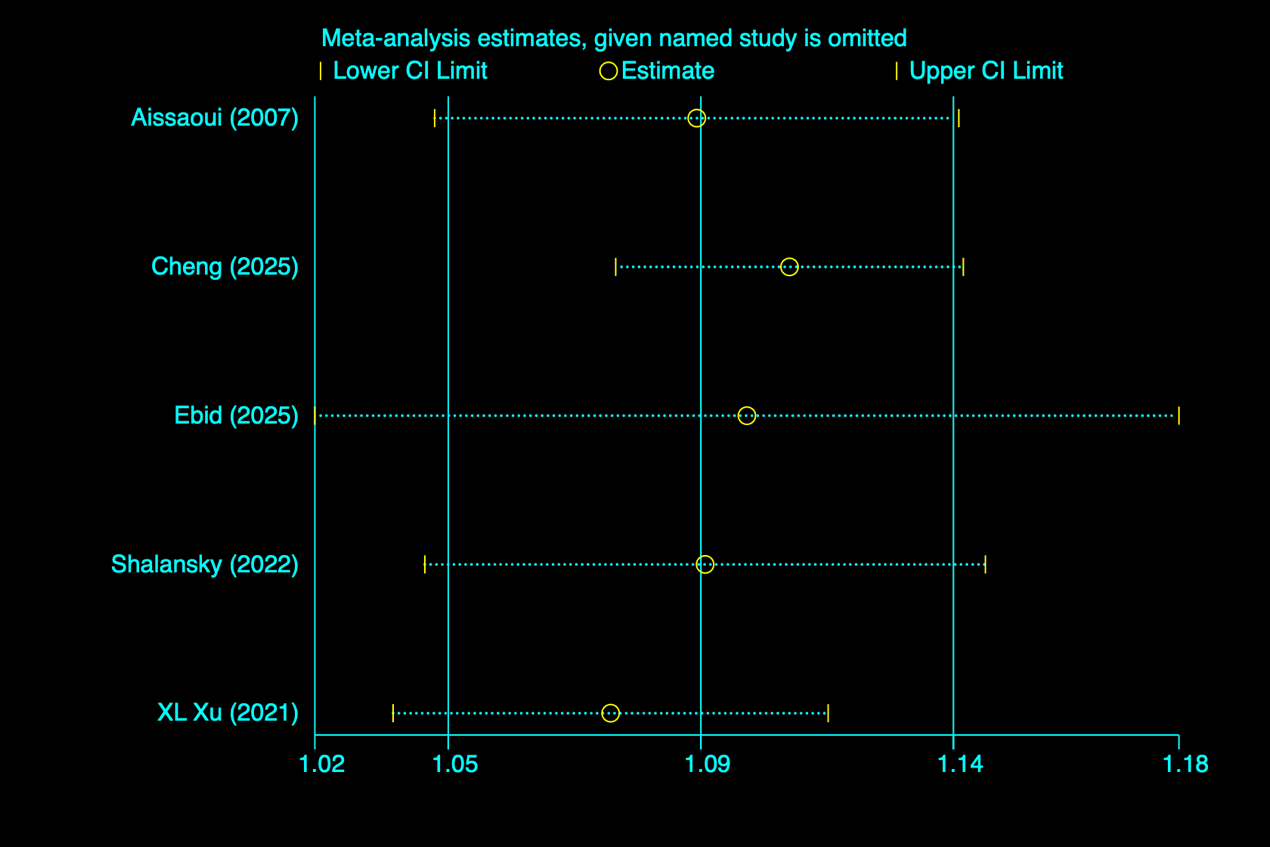


Figure S3 sensitivity analysis results of Simplified Acute Physiology Score (SAPS) < 20


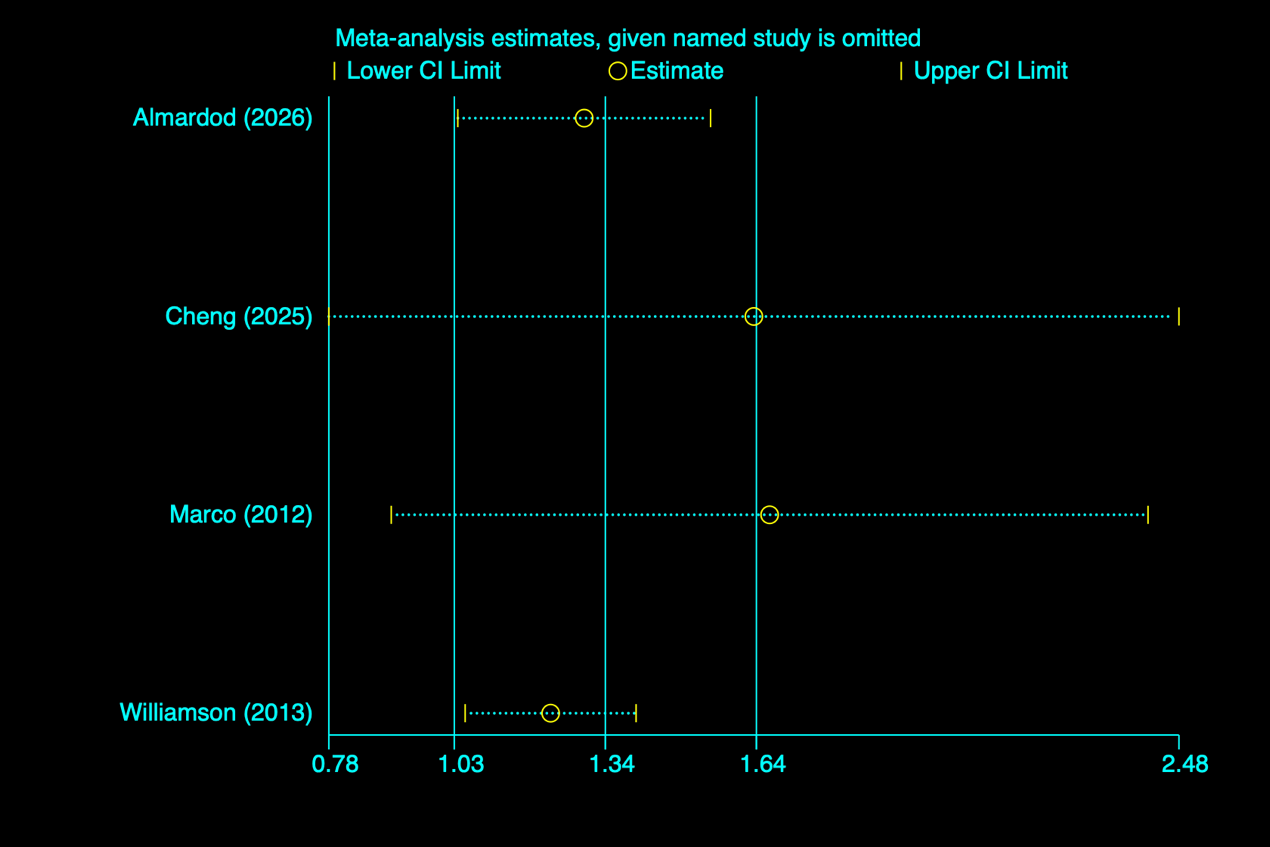


Figure S4 sensitivity analysis results of impaired liver function


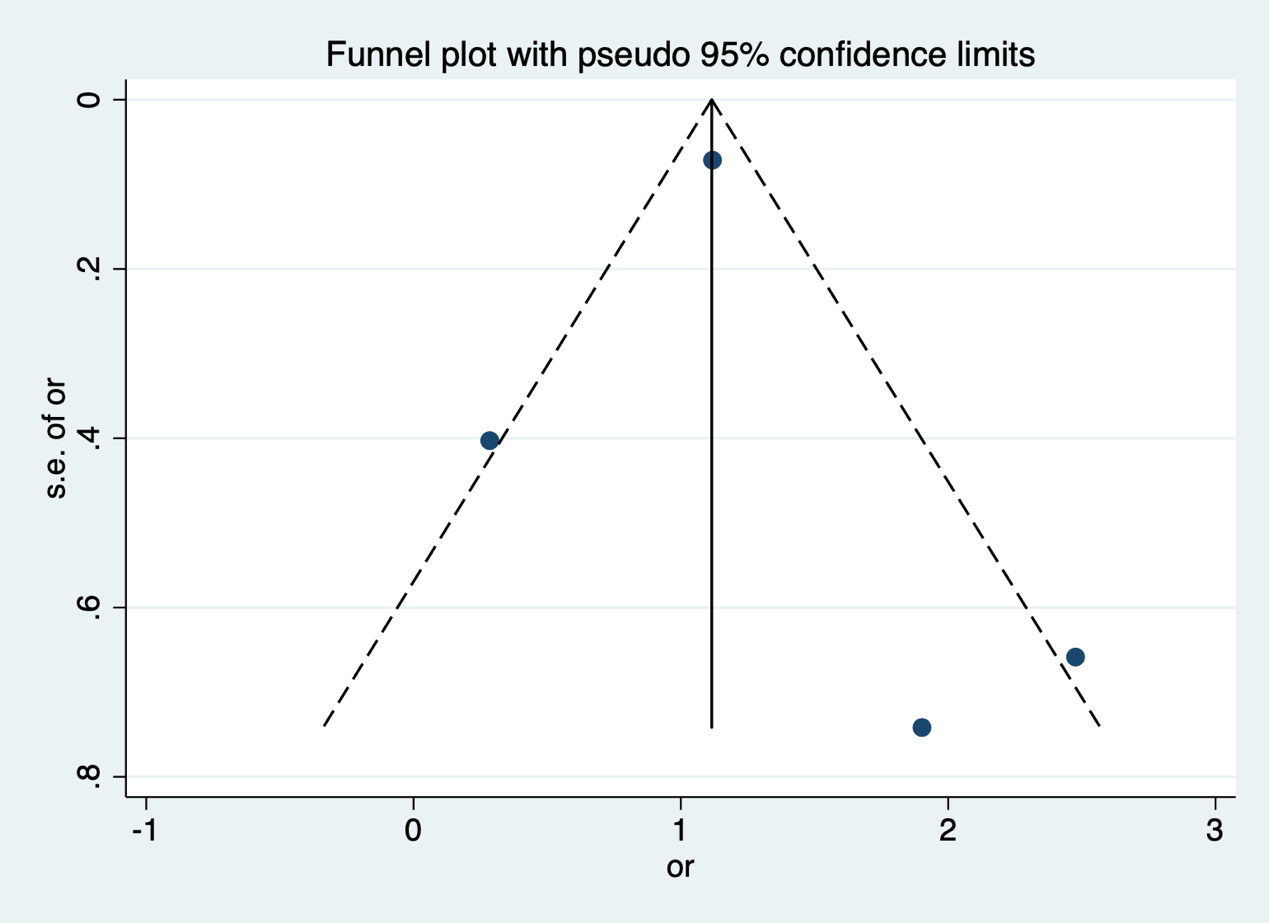


Figure S5 Funnel plot of the meta-analysis of bleeding


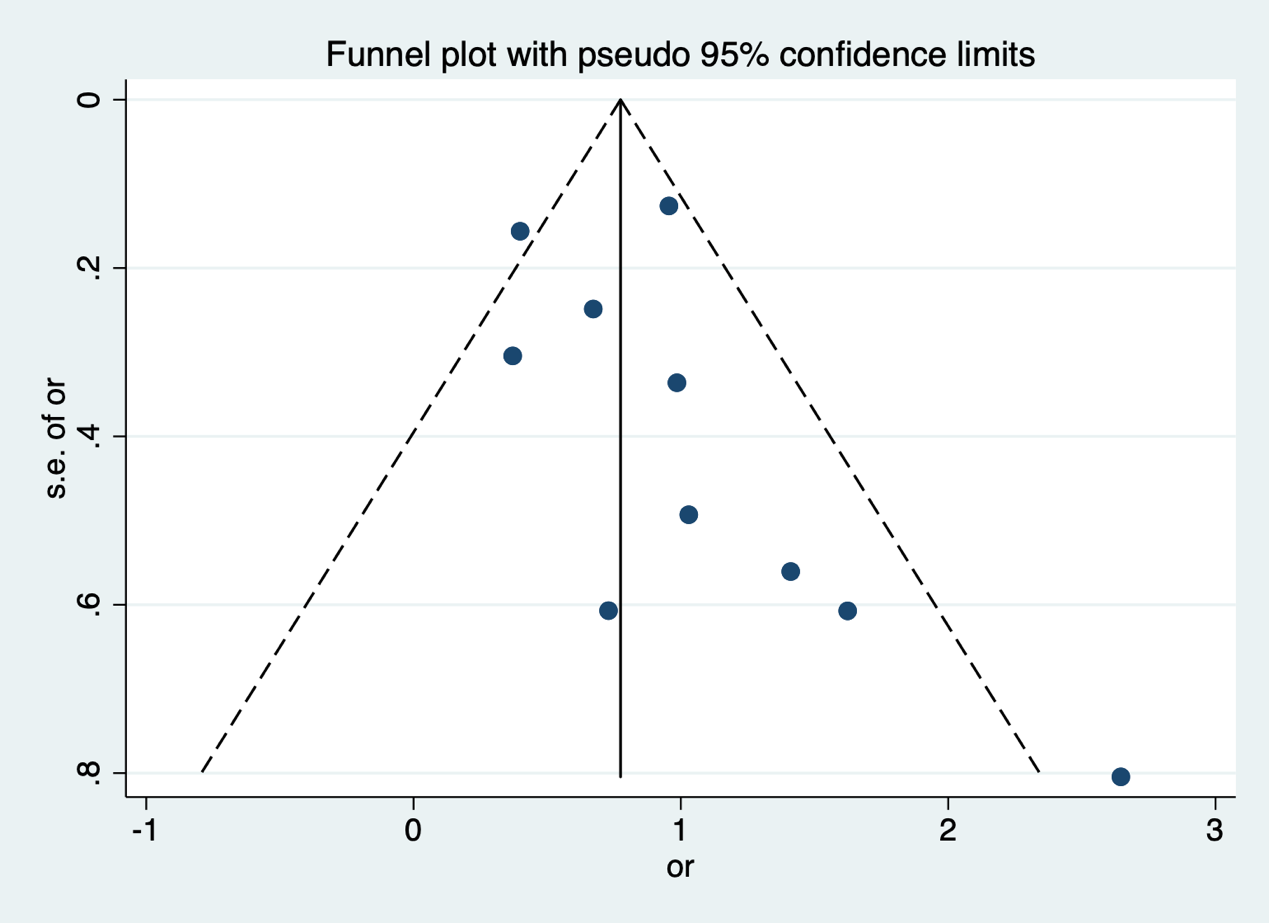


Figure S6 Funnel plot of the meta-analysis of sepsis


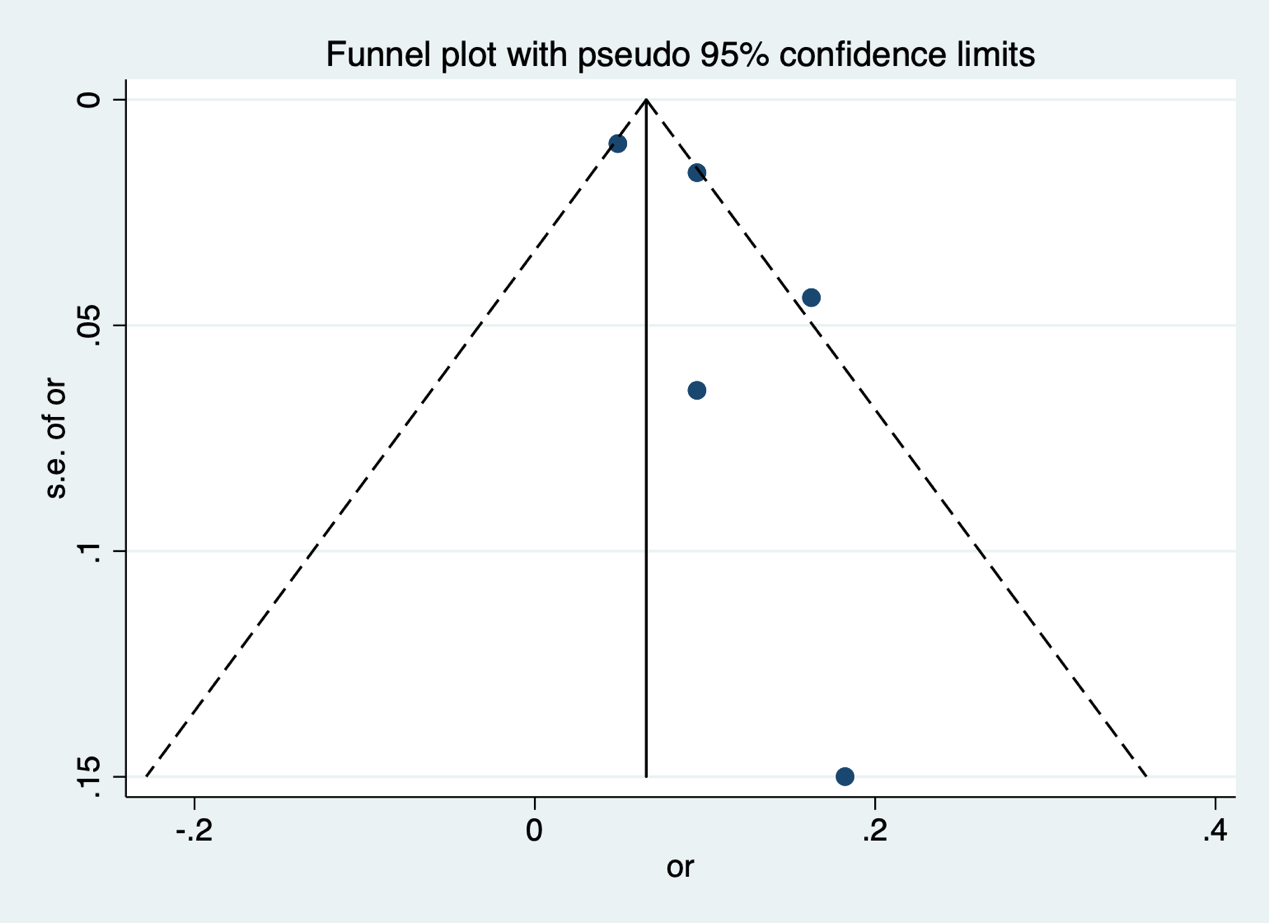


Figure S7 Funnel plot of the meta-analysis of SAPS< 20


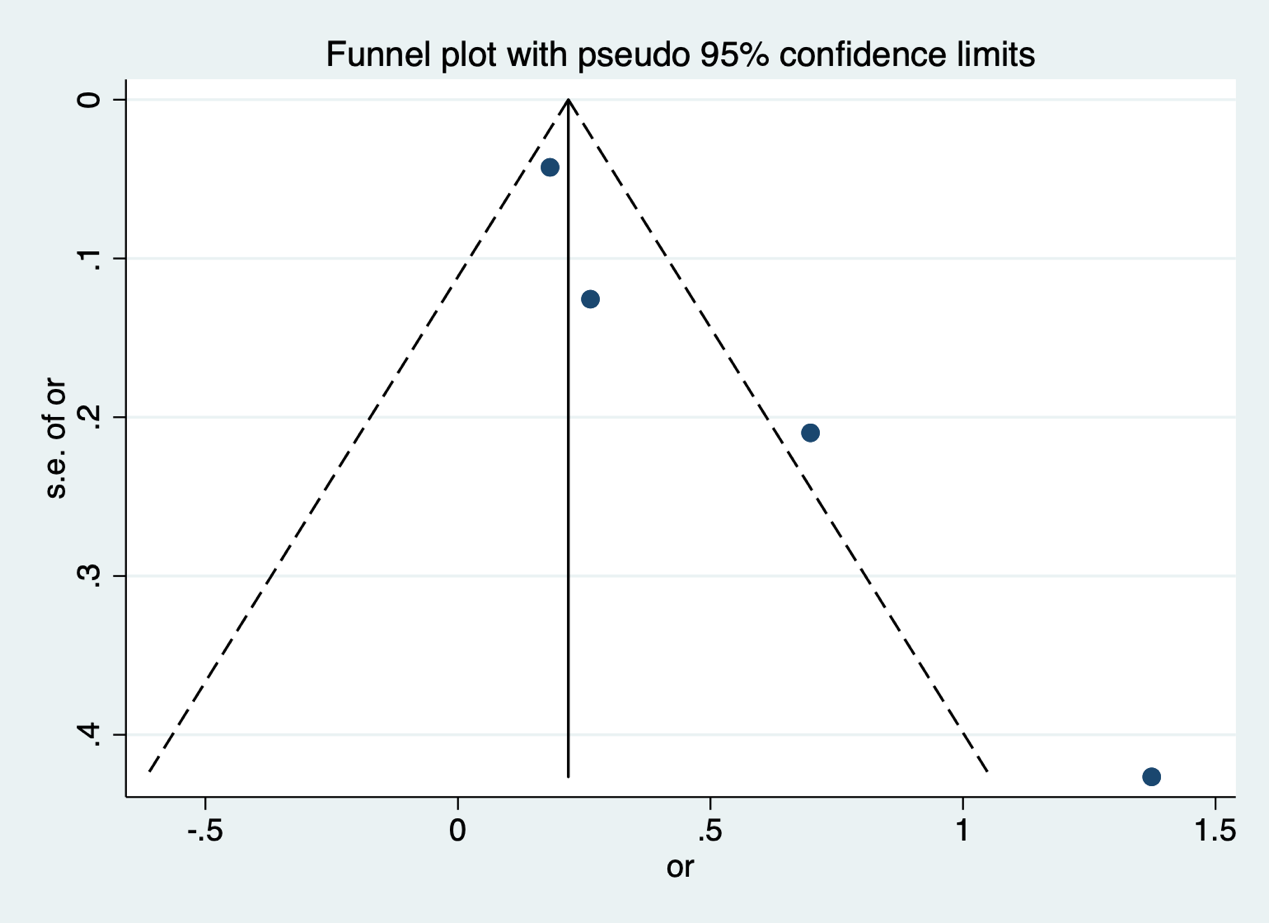


Figure S8 Funnel plot of the meta-analysis of impaired liver function


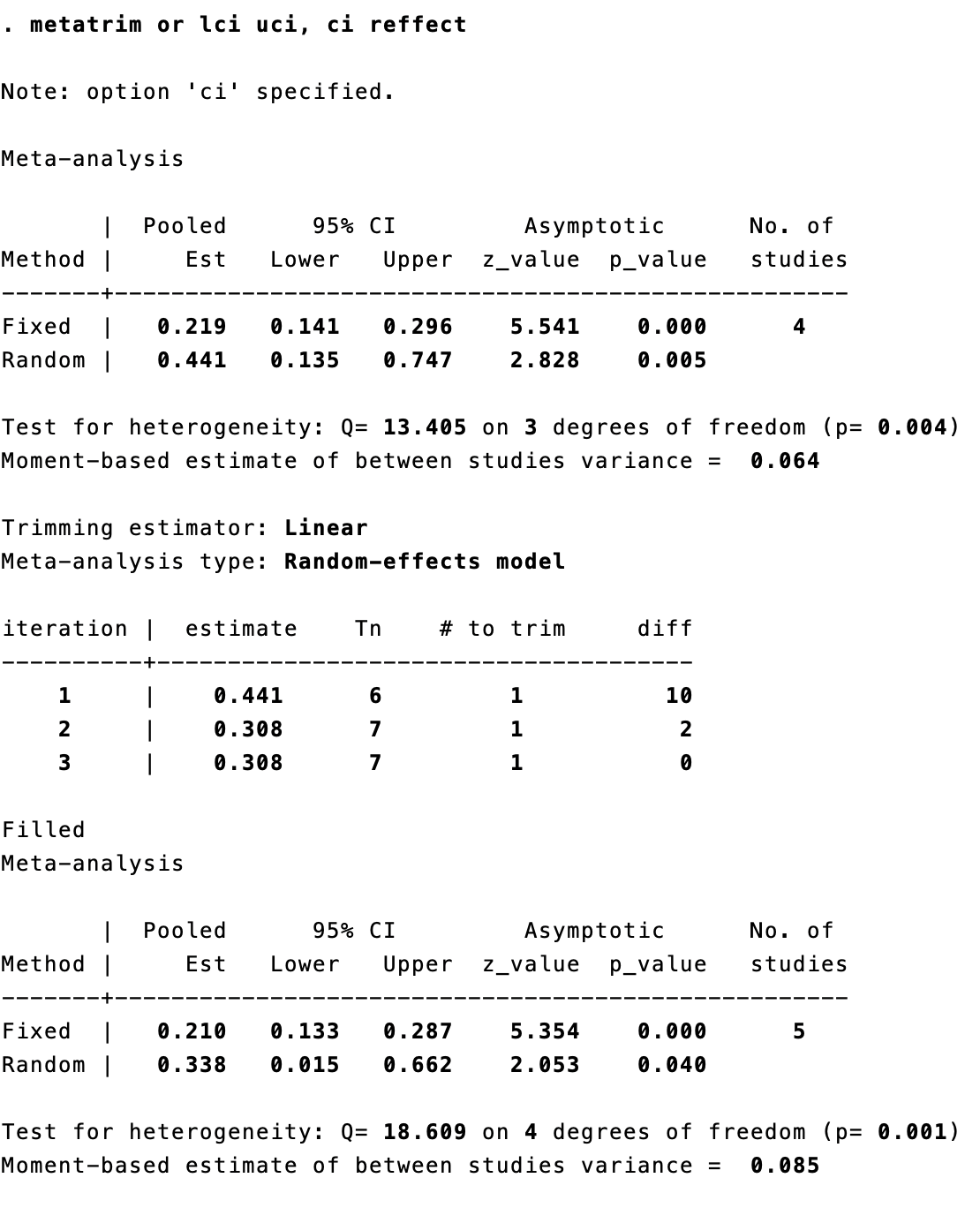


Figure S9 trim-and-fill results of impaired liver function
